# Structural Flexibility in Metal-Organic Cages

**DOI:** 10.3389/fchem.2021.706462

**Published:** 2021-06-17

**Authors:** Andrés E. Martín Díaz, James E. M. Lewis

**Affiliations:** Department of Chemistry, Imperial College London, Molecular Sciences Research Hub, London, United Kingdom

**Keywords:** self-assembly, cages, flexibility, host-guest, metallosupramolecular

## Abstract

Metal-organic cages (MOCs) have emerged as a diverse class of molecular hosts with potential utility across a vast spectrum of applications. With advances in single-crystal X-ray diffraction and economic methods of computational structure optimisation, cavity sizes can be readily determined. In combination with a chemist’s intuition, educated guesses about the likelihood of particular guests being bound within these porous structures can be made. Whilst practically very useful, simple rules-of-thumb, such as Rebek’s 55% rule, fail to take into account structural flexibility inherent to MOCs that can allow hosts to significantly adapt their internal cavity. An often unappreciated facet of MOC structures is that, even though relatively rigid building blocks may be employed, conformational freedom can enable large structural changes. If it could be exploited, this flexibility might lead to behavior analogous to the induced-fit of substrates within the active sites of enzymes. To this end, in-roads have already been made to prepare MOCs incorporating ligands with large degrees of conformational freedom. Whilst this may make the constitution of MOCs harder to predict, it has the potential to lead to highly sophisticated and functional synthetic hosts.

## Introduction

Metal-organic cages (MOCs) are self-assembled structures derived from carefully selected combinations of metal ions and ligands (Cook and Stang, 2015; Debata et al., 2019; Pilgrim and Champness, 2020). Porous MOCs of myriad shape and size are capable of binding guest molecules in their cavities (Rizzuto et al., 2019), leading to applications in catalysis (Yoshizawa et al., 2009; Sinha and Mukherjee, 2018), storage of reactive species (Galan and Ballester, 2016), molecular separations (Zhang et al., 2021), and drug delivery (Casini et al., 2017) amongst others. Guest-binding is a complex process involving contributions from enthalpic and entropic factors for both the encapsulation of the guest(s) and the liberation of solvent molecules (or displacement of extant guest molecules) (Metherell et al., 2018). The 55% rule originally established by Rebek (Mecozzi and Rebek, 1998) has been a good guiding principle when predicting molecular binding based on the packing coefficient of the guest inside the host’s cavity. This volume-based approach, however, assumes the cavity to be static and therefore does not take into consideration host flexibility, which can greatly affect cavity size and shape and is a relatively underexplored consideration. The ability to harness this structural flexibility could lead to enzyme-like conformational adaptability (Boehr et al., 2009; Feixas et al., 2014). Characterising host flexibility is therefore crucial to understanding its effects on any potential applications.

In this mini review we highlight examples from the literature that demonstrate the inherent structural flexibility of *rigid* metal-organic hosts, both in solution and the solid-state, and examine the limited number of examples wherein units with high degrees of conformational freedom have been purposefully incorporated into ligand frameworks. A focus is placed on assemblies that undergo conformational, rather than configurational, changes without requiring dissociation and rearrangement of components. Full-scale compositional changes will not be covered, although it is noted than for some examples the involvement of bond dissociation in the mechanism of observed adaptability cannot be absolutely excluded. Neither will assemblies with stimuli-responsive, switchable units be included (Han et al., 2013; Oldknow et al., 2018), which have been covered elsewhere (McConnell et al., 2015). This review is not intended to be comprehensive—selected examples from the literature have been chosen to demonstrate key principles.

## Observation of Structural Flexibility in Solution

Various 1D and 2D NMR techniques are commonly used to characterise MOCs and to probe their guest-binding properties. Flexibility arising from conformational freedom, however, is likely to occur rapidly on the NMR timescale, resulting in a time-averaged structure. This makes it difficult to gain accurate information regarding the extent of conformational plasticity. Despite this, observations made during particular experiments can allow inference of structural flexibility, often aided by use in conjunction with SCXRD data.

**FIGURE 1:**
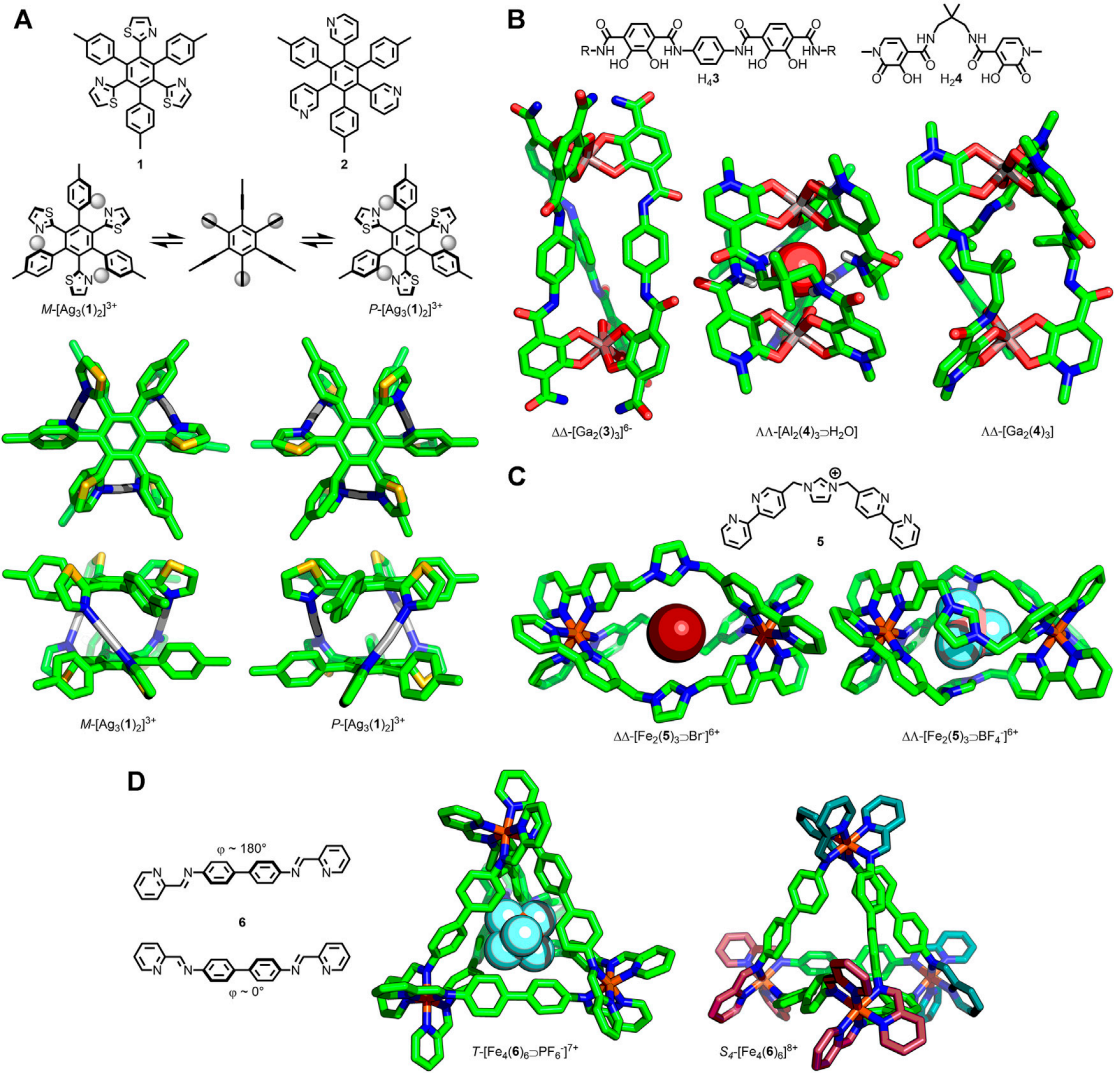
**(A)** Shionoya’s trinuclear *P*/*M*-[Ag_3_(**1**/**2**)_2_]^3+^ helicates capable of undergoing stereochemical inversion *via* an achiral intermediate. **(B)** Raymond’s [Ga_2_(**3**)_3_] helicate able to undergo helical inversion *via* a Bailar twist mechanism; SCXRD structures of [Al_2_(**4**)_3_⋑H_2_O] helicate and [Ga_2_(**4**)_3_] mesocate. **(C)** Wu’s triply-stranded [Fe_2_(**5**)_3_⋑X^−^]^6+^ assemblies with encapsulated anion-dependent formation of helicate (X^−^ = Br^−^) or mesocate (X^−^ = BF_4_^−^) structures. **(D)** Nitschke’s [Fe_4_(**6**)_6_]^8+^ tetrahedra, with encapsulation of a PF_6_^−^ anion favouring the *T*-symmetric assembly, whilst the *empty* cage preferentially crystallised as the *S*_*4*_ structure with ∆ (cyan) and *Λ* (magenta) Fe(II) centers.

In 2003 Shionoya and co-workers reported the synthesis of the helical sandwich-shaped assembly [Ag_3_(**1**)_2_]^3+^ as a racemic mixture of the *M* and *P* isomers (Figure 1A), demonstrated in solution through the formation of the Δ-TRISPHAT salt (Hiraoka et al., 2003). At 253 K (in 2:1 *d*_6_-acetone/CDCl_3_) two sets of equal intensity signals were observed for the two enantiomers, suggesting a lack of chiral induction from the anion. Upon increasing the temperature to 303 K coalescence to a single set of signals was observed, the result of rapid interconversion between the *P* and *M* enantiomers on the NMR timescale. In order to switch between the two enantiomers, the cage likely transitions through a high energy, achiral conformer with an increased distance between the core phenyl units of the ligands (< 1 Å). In an analogous complex, [Ag_3_(**2**)_2_]^3+^, interconversion between isomers occurred rapidly only at elevated temperatures, indicating a larger energy barrier to helical inversion.

Raymond and co-workers investigated the stereochemical inversion of triply-stranded dinuclear helicates assembled from bis-catecholate ligands (**3**) and Ga(III) ions (Figure 1B) (Kerstin et al., 1996). From a comprehensive NMR study the authors were able to ascertain that the isomers were interconverting through an intramolecular helical inversion process (ΔΔ⇋ΛΛ), with independent chiral inversion occurring at each metal centre through a non-dissociative Bailar twist mechanism, *via* a mesocate intermediate. Subsequent work with related [M_2_(**4**)_3_] [M = Al(III) or Ga(III)] assemblies (Figue 1B) demonstrated an ability to controllably switch between the chiral helicate (M···M 7.13 Å) and larger, achiral mesocate (M···M 9.74 Å) using host-guest interactions (Xu et al., 1999). Despite crystallising as the mesocate, [Ga_2_(**4**)_3_] existed in solution as an equilibrium between the helicate and mesocate forms. The helicate was shown to be stabilised by an encapsulated solvent molecule (water or DMSO); increasing the water concentration served to shift the equilibrium further towards the helicate.

More recently Wu and co-workers (Cui et al., 2012) observed a similar change in the structure of a triply-stranded, dinuclear structure, assembled from bis-bidentate ligand **5** and Fe(II), with different anions bound in its cavity (Figure 1C). With Br^−^ or NO_3_^−^ encapsulated a helicate assembly was formed. In contrast, the tetrahedral anions BF_4_^−^, ClO_4_^−^, and SO_4_^2−^ resulted in mesocates, with the helicates being significantly longer (Fe···Fe ∼11.7 Å vs ∼10.6 Å). This switch between helicate and mesocate was reasoned to arise from conformational changes of the ligand in order to maximize favourable interactions with the encapsulated anion. In the case of the mesocate the ligands adopt a C-shaped conformation, whilst an S-conformer led to the helicate assembly.

A related concept has been studied by Nitschke and co-workers, wherein the relative stereochemistry of octahedral metal centres within a tetrahedral MOC could be affected by encapsulation of different anions, impacting the overall symmetry of the assembly (Clegg et al., 2013). Subcomponent self-assembly of 4,4′-diaminobiphenyl, 2-formylpyridine and Fe(II) with large triflimide counteranions resulted in a tetrahedral cage structure, [Fe_4_(**6**)_6_]^8+^ (Figure 1D). In the solid state, SCXRD data demonstrated that the cage possessed two metal centres of one stereochemistry and two of the opposite (i.e., ΔΔΛΛ), giving the tetrahedron an overall *S*_4_ symmetry [with two ligands adopting an S-shaped conformation (*φ* ≈ 180°), the remaining four a C-shape ( *φ* ≈ 0°)]. In solution, however, a mixture of *S*_4_-, *T*-, and *C*_3_-symmetric assemblies were observed in an approximately 5:3:2 ratio at equilibrium. Smaller anions were capable of binding within the cavity of the cage, and altering the equilibrium position of this diasteroeomeric mixture, with ClO_4_^−^ and PF_6_^−^ shifting the equilibrium in favor of the *T*-symmetric isomer (ΔΔΔΔ/ΛΛΛΛ; all ligands S-conformation). Remarkably NO_3_^−^, BF_4_^−^, Cl^−^, Br^−^, and I^−^ encapsulation all resulted in quantitative conversion to the *T* diasteroisomer, as determined by solution-phase NMR analysis. In addition, SCXRD data demonstrated that the MOC framework was able to conformationally adapt to encapsulate the various anions; the cavity volume was found to expand from 69 Å^3^ with BF_4_^−^ to 87 Å^3^ with PF_6_^−^.

In these studies solution phase data were able to demonstrate structural changes in the MOCs arising from alterations to the assembly’s symmetry. More subtle changes from, e.g., flexing and conformational adjustment of ligands, are not so readily detected in this manner. SCXRD, however, provides more precise structural information and was used effectively in combination with the solution-phase data in the previous examples to add quantitative spatial information. It is important to note that the solid-state data merely provides a *snap-shot* image of the assemblies, which can be affected by various packing interactions. Multiple sets of SCXRD data, however, can show differences in structural parameters, allowing inference of the extent of MOC flexibility.

## Solid-State SCXRD Evidence for Structural Flexibility

**FIGURE 2:**
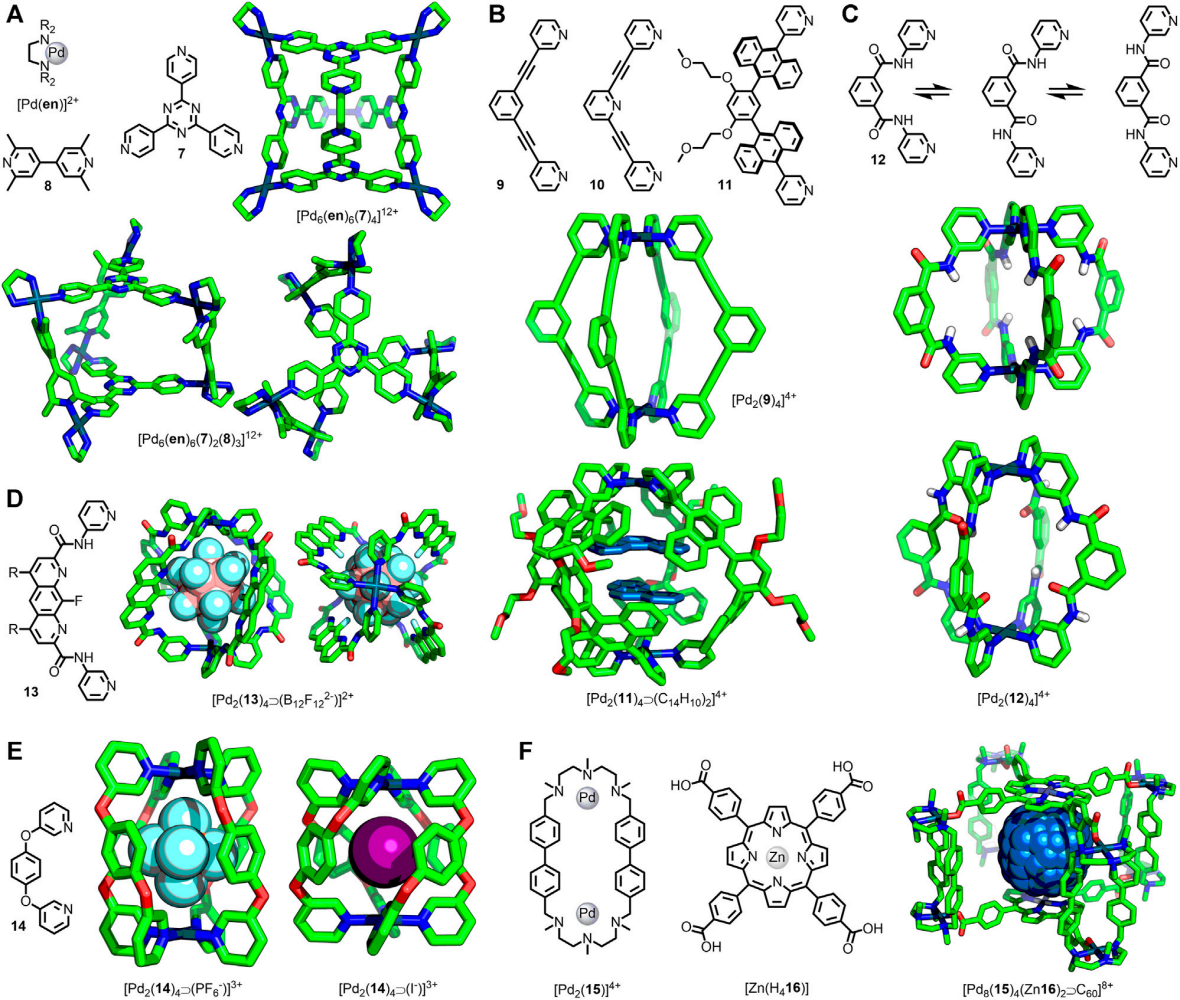
**(A)** Fujita’s octahedral cage, [Pd_6_(**en**)_6_(**7**)_4_]^12+^, and trigonal prismatic assembly [Pd_6_(**en**)_6_(**8**)_3_(**7**)_2_]^12+^ shown from the side and above. **(B)** Ditopic ligands **9**, **10** and **11** used in the assembly of Pd_2_L_4_ cages. SCXRD structures of [Pd_2_(**9**)_4_]^4+^ and [Pd_2_(**11**)_4_⋑(C_14_H_10_)_2_]^4+^. **(C)** Puddephatt’s ditopic ligand **12** with three possible conformations accessed through rotation about the amide bonds; SCXRD structures of [Pd_2_(**12**)_4_]^4+^ with **12** exhibiting two different ligand conformations. **(D)** Gan’s [Pd_2_(**13**)_4_⋑(B_12_F_12_^2-^)]^2+^ cage viewed from the side **(left)** and above **(right)**. **(E)** McMorran and Steel’s [Pd_2_(**14**)_4_]^4+^ helicates with PF_6_^−^ **(left)** and I^−^ **(right)** anions bound. **(F)** Ribas’ porphyrin-based cage, [Pd_8_(**15**)_4_(Zn**16**)_2_]^8+^, with encapsulated C_60_ guest.

Arguably one of the most studied MOCs in the literature is the hexanuclear octahedron ([Pd_6_(**en**)_6_(**7**)_4_]^12+^; Figure 2A) originally reported by the Fujita group (Fujita et al., 1995). For this assembly multiple sets of SCXRD data are available with different guests encapsulated within the host, allowing quantitative comparison of structural parameters in the solid state (Tashira et al., 2005; Nishioka et al., 2007; Kohyama et al., 2014; Takezawa et al., 2014; Cullen et al., 2019; Takezawa et al., 2020). These demonstrate that the M···M distance varies by less than 1% across numerous solid-state structures. Clearly, in this instance, significant distortion of the cage structure through host-guest interactions and crystal packing effects is not in evidence. For other MOCs, however, such effects have been observed.

The heteroleptic trigonal prismatic cage [Pd_6_(**en**)_6_(**7**)_2_(**8**)_3_]^12+^ (Figure 2A), also reported by Fujita and co-workers (Yoshizawa et al., 2005a), was assembled from **7** and 2,2′-6,6′-tetramethyl-4,4′-bipyridine (**8**) with *cis*-Pd^II^(**en**) metal nodes (Figure 1A). Due to the manner in which the ligands are arranged in this assembly, **7** and **8** are orthogonal in the most symmetric form. In this conformer the two **7** ligands would eclipse one another and the torsion angle between them would be 0°. As this angle increases, the **7** ligands will become more offset, and the angle between **7** and **8** will diverge from orthogonality. With extensive investigation of this cage’s host-guest properties having been undertaken, it was possible to examine multiple SCXRD structures of the assembly to analyse changes in structural parameters. From this it was seen that the average torsion angle varied between ∼19 and ∼39°, and the distance between triazines ranged from 9.9–10.6 Å (Yoshizawa et al., 2005a; Yoshizawa et al., 2005b; Ono et al., 2007; Ono et al., 2009; Yamauchi et al., 2010; Schmidt et al., 2016).

Torsional twisting of trigonal prismatic cages, leading to a dramatic change in cavity size, has been reported by others. Severin and co-workers prepared a hexanuclear cage from ligand **7** and diruthenium(II) clips. Upon binding two molecules of coronene in the cavity the torsion angle between the two **7** ligands decreased from ∼75–∼3°, increasing the distance between them from 3.4–11.1 Å, thereby expanding the cavity size from effectively zero to > 500 Å^3^ (Mirtschin et al., 2010). Likewise, Han and Jin have reported a trigonal prism assembled from **7** and diiridium(III) clips that collapsed (**7**···**7** distance reduced from 6.9–3.3 Å) through increased torsion between the **7** ligands (∼16–∼30°) upon removal of a coronene guest molecule (Han and Jin, 2011).

Ditopic *banana-shaped* ligands (Han et al., 2014) are commonly combined with Pd(II) ions to form dinuclear, lantern-shaped architectures. These are often designed with the ligand framework fully conjugated; however, rotation about certain bonds is still feasible, potentially introducing some dynamic character to the resultant assemblies. In 2010 Hooley and co-workers reported the cage [Pd_2_(**9**)_4_]^4+^ (Figure 2B) and its SCXRD structure with an encapsulated OTf^−^ anion (Liao et al., 2010). More recently, Lusby and co-workers have examined this cage for its catalytic potential (Spicer et al., 2020) and were able to obtain SCXRD data of the PF_6_^−^ salt with pentacenedione bound in the cavity (August et al., 2016). Although relatively minor, there was a difference of 0.36 Å in the Pd···Pd distance between the cage SCXRD structures (11.85 and 12.21 Å), and the torsion angle between the core phenyl and terminal pyridyl rings were greater for the longer cage (12.5° on average compared to 5.1°).

The related [Pd_2_(**10**)_4_]^4+^ architecture, in which the central phenyl unit of the ligand is replaced with a pyridine, has been reported by Crowley and co-workers. They were able to obtain SCXRD structures of various salts of the cage (Lewis and Crowley, 2014), as well as a host-guest adduct with two cisplatin molecules (Lewis et al., 2012). Again, some variation in the Pd···Pd distance was observed (11.5–12.2 Å) with torsion angles between the core and coordinating pyridine rings ranging from 2.5–19.0°.

In a similar Pd_2_L_4_ system (Figure 2B) reported by Yoshizawa and co-workers (Kishi et al., 2011), with anthracene units linking the core phenyl ring and coordinating pyridines instead of alkynes (ligand **11**), even more dramatic differences were observed with various host-guest adducts. For instance, in the 1:1 adduct formed between the cage and [2.2]-paracyclophane the Pd···Pd distance was found to be 13.8 Å. In contrast, with two molecules of corranulene encapsulated this was found to shrink to 12.4 Å, a reduction of 10% (Kishi et al., 2013).

Puddephatt and co-workers prepared ligand **12** with 3-pyridyl units attached to a *meta*-phenyl core through amide bonds. Rotation about these amide bonds ostensibly permitted three conformations of the ligand (Figure 2C). When combined with Pd(II) ions the expected [Pd_2_(**12**)_4_]^4+^ assembly was obtained, wherein adoption of different ligand conformations could alter the character of the cavity depending on whether the amide carbonyl or N-H units were directed internally. SCXRD structures of the cage (Figure 2C) with both F_3_CSO_3_^−^ and F_3_CCO_2_^−^ anions were obtained. In the case of the former both N-H units of each ligand were directed endohedrally, allowing the formation of hydrogen-bonds to partially encapsulated F_3_CSO_3_^−^ anions. Conversely the F_3_CCO_2_^−^ salt resulted in adoption of ligand conformations with one carbonyl directed exohedrally, the other endohedrally acting as a hydrogen-bond acceptor to encapsulated water molecules. This change from a higher to lower symmetry assembly was coincident with a lengthening of the Pd···Pd distance from 9.5–11.2 Å (Yue et al., 2003). Despite its lower symmetry in the solid state, the observation of a single set of signals for the pyridyl units by NMR demonstrated rapid conformational interconversion on the NMR timescale (Yue et al., 2004).

Likewise, Gan and co-workers were able to observe significant changes in structural parameters in a similarly designed system (Lin et al., 2018). [Pd_2_(**13**)_4_]^4+^ (Figure 2D) was assembled from ligand **13** and an appropriate Pd(II) source. SCXRD structures for this architecture were obtained with BF_4_^−^, NO_3_^−^ (two polymorphs were reported), and B_12_F_12_^2−^ anions encapsulated within the internal cavity, with changes in the ligand conformation demonstrated through significant variation in the Pd···Pd distance (12.1–13.4 Å).

Unsurprisingly the introduction of saturated units within the ligand framework offers the potential for greater degrees of flexibility within assemblies. In 1998 McMorran and Steel reported the first example of a dipalladium(II) helical cage assembled from a ditopic ligand, namely 1,4-bis(3-pyridyloxy)benzene (**14**) (McMorran and Steel, 1998). In contrast to the previous examples, the ether linkage between the core and coordinating units engendered the ligands with more significant degrees of freedom. This work was recently extended to include a thorough examination of the anion-binding properties of this cage, including an extensive study in the solid state from SCXRD data (Steel and McMorran, 2019). This revealed a significant 16% reduction in the Pd···Pd distance upon exchanging a bound PF_6_^−^ anion (8.84 Å) for an I^−^ guest (7.44 Å) (Figure 2E), the result of a drastic change in the Pd-N···N-Pd torsion angle (average 41.5° for PF_6_^−^ and 80.9° for I^−^). The associated reduced helical pitch (77–37 Å) reduced the effective cavity size from 92–41 Å^3^.

## Molecular Dynamics Simulations of Metal-Organic Cage Flexibility

Computational modelling of supramolecular systems has become cheaper and more readily available in recent years, allowing combinations of experimental and theoretical data to be used to gain in-depth understanding of these artificial mimics of biological assemblies (Frederix et al., 2018). Given the previously mentioned constraints placed on information related to conformation dynamics that can be gleaned from solution and solid-state data, computational analysis offers a potential route to quantitatively examine flexibility of metal-organic assemblies. Perhaps most interestingly these techniques could be effectively used to aid in the design of such systems for specific applications (Young et al., 2020a).

Recently Lusby, Duarte and co-workers utilised molecular dynamics (MD) simulations to explore the conformational flexibility of the two dipalladium(II) cages assembled from **9** and **10** to explain observations related to their catalytic activity (Young et al., 2020b). In these simulations the Pd···Pd distance in the cages was found to vary between 11.3 and 12.9 Å for ligand **9**, and 10.9 and 12.5 Å for ligand **10**, with the torsion angle between the core ring and terminal pyridines ranging from 0–60°. Interestingly, for the [Pd_2_(**10**)_4_]^4+^ cage, conformations with significant twisting of the ligands were found to be of similar energy to the highly symmetric structure (conformer with *Φ* = 41° was only 2.6 kcal mol^−1^ higher in energy than the symmetric *Φ* = 0° conformer).

Ribas and co-workers reported the synthesis of a nanocage assembled from four dinuclear macrocycle Pd(II) complexes ([Pd_2_(**15**)]^4+^) and two tetracarboxylate Zn(II)-porphyrins ([Zn(H_4_ **16**)]) (García-Simón et al., 2014). These cages were observed to bind fullerene guests, and the SCXRD structures of the “empty” cage and C60 and C70 adducts were obtained (Figure 2F). For the empty cage the distance between Zn(II) ions was 14.1 Å, with a contraction seen for the C70 (13.8 Å) and C60 (13.1 Å) host-guest adducts. This was concomitant with a small decrease in the Pd···Pd distance within the macrocycles from 11.25–11.17 Å. Subsequent molecular dynamics simulations (2.5 μs) showed flexing of the cage to give Zn···Zn distances ranging between 11.3 and 15.8 Å. Binding of fullerene guests induced a compression of this distance, consistent with the SCXRD structures. To enable fullerene guests to enter the cage cavity a transient expansion of both the portal size, from ∼50 Å^2^ up to ∼90 Å^2^, and the Zn···Zn distance (∼15 Å) was observed, demonstrating a significant dynamic range for this host assembly (García-Simón et al., 2020).

## Metal-Organic Assemblies Derived From Ligands With Large Amplitude Conformational Flexibility

The previous sections have sought to demonstrate that even MOCs assembled from what would normally be considered rigid ligands–including those with fully conjugated systems–can display a reasonable amount of flexibility. There is a paucity of examples in the literature, however, of purposeful introduction of units that display large-amplitude conformational freedom into metal-organic systems. A key facet of metallo-supramolecular self-assembly is predicting products of self-assembly based on constituent metal ion geometry and ligand conformation. The importance of ligand conformation has been amply demonstrated by the work of the Fujita group on Pd_*n*_L_*2n*_ cages assembled from ditopic ligands, in which the ligand angle determines the thermodynamically favoured value of *n* (Harris et al., 2013). When multiple ligand conformers are accessible, predicting/controlling self-assembly outcomes becomes more difficult. Adaptability, however, is a hallmark of natural self-assembled systems, and could offer an increased level of stimuli-responsiveness if incorporated into artificial systems seeking to mimic Nature’s success.

**FIGURE 3:**
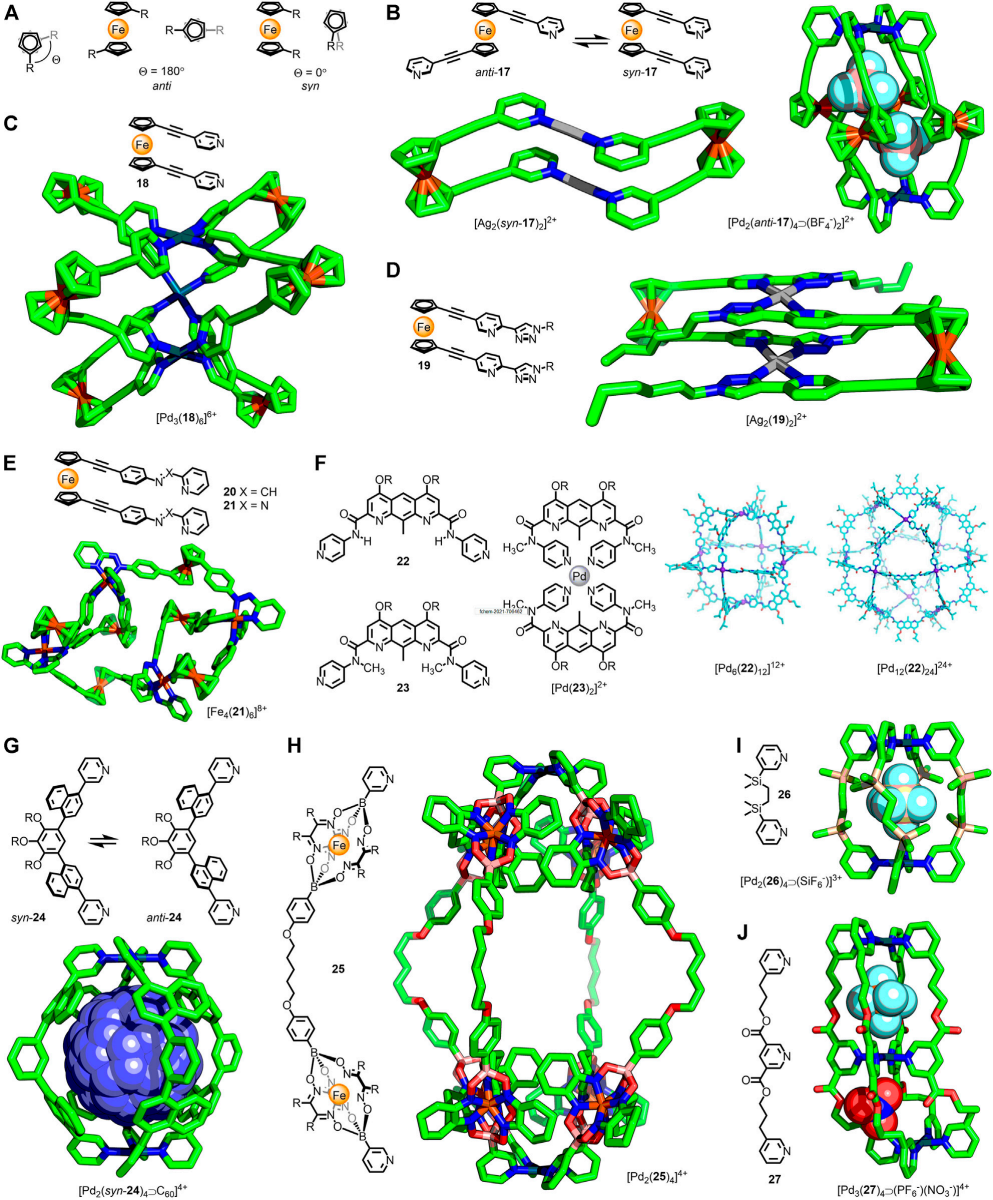
**(A)** The cyclopentadiene rings of ferrocene are able to undergo 360° rotation. The two extremes of conformation are the *anti* (*Θ* = 180°) and *syn* (*Θ* = 0°) conformers. **(B)** In the dinuclear metallacycle [Ag_2_(**17**)_2_]^2+^ ligand **17** is observed to adopt a *syn* conformation; in the quadruple-stranded [Pd_2_(**17**)_4_]^4+^ cage the *anti* conformer is observed. **(C)** Crowley’s trigonal prismatic [Pd_3_(**18**)_6_]^6+^ assembly with ligands in *syn* arrangements (*Θ* = 25–42°). **(D)** A dinuclear metallacycle, [Ag_2_(*syn*-**18**)_2_]^2+^ reported by Crowley. **(E)** Nitschke’s twisted parallelogram assembly, with ligand **21** in both *syn* and *anti* conformations. **(F)** Non-covalent interactions affect favoured ligand conformations: self-assembly with Pd(II) led to a Pd_12_L_24_ cuboctahedron with **22** (*via* a kinetic Pd_6_L_12_ cube; calculated structures shown), whilst ligand **23** gives the mononuclear [Pd(**23**)_2_]^2+^ complex. Adapted with permission from Liu et al. (2020). Copyright © 2020 Wiley-VCH GmbH. **(G)** Yoshizawa’s atropisomeric ditopic ligand that formed [Pd_2_(**24**)_4_]^4+^ assemblies; guest encapsulation biased the distribution of cage atropisomers. **(H)** Severin’s alkyl-linked metallo-ligand that formed the large (Pd···Pd ∼ 3 nm) [Pd_2_(**25**)_4_]^4+^ cage. **(I)** Alkyl-linked ligand **26** formed Pd_2_L_4_ cages capable of anion-encapsulation. **(J)** Jung’s trinuclear, double-cavity cage bound anions in each cavity, which adapt their shape to best match the encapsulated guest.

Ferrocene, first synthesised 70 years ago (Kealy and Pauson, 1951), is the most well-known of the organometallic sandwich complexes. The ability of the two cyclopentadiene ligands to rotate relative to each other has led to significant interest in ferrocene’s use as a “ball-bearing” component in molecular machines (Scottwell and Crowley, 2016). The ability of the ligands to undergo a full 360° rotation means that the dihedral angle between substituents of 1,1′-disubstituted ferrocenes can vary from 0 (*syn*)–180° (*anti*) (Figure 3A).

In 2003 Lindner and co-workers reported the self-assembly of 1,1′-bis(pyridin-3-ylethynyl)ferrocene (**17**) with Ag(I) and *trans*-Pd(II)Cl_2_ metal nodes (Lindner et al., 2003). In both instances dinuclear metallacycles were obtained (Figure 3B), with the ligands adopting a stacked conformation (*Θ* = 7°). Crowley and co-workers subsequently examined the self-assembly of this ligand with Pd(II) ions (Vasdev et al., 2019), observing formation of [Pd_2_L_4_]^4+^ cages (Figure 3B) with the ligands in *anti* conformations (*Θ* = 105–134°). SCXRD structures of the cage revealed that the Pd···Pd distance was affected by changes in the dihedral angle between the pyridyl arms (13.1 Å for one crystallographically independent molecule of [Pd_2_(**17**)_4_⋑(BF_4_^−^)_2_]^2+^ and *Θ*_*ave*_ = 109°; 13.4 Å for [Pd_2_(**17**)_4_⋑(SbF_6_^−^)]^3+^ and *Θ*_*ave*_ = 126°), demonstrating an ability for the cage to adapt its shape based on the ligand conformation.

Self-assembly of the isomeric 1,1′-bis(pyridin-4-ylethynyl)ferrocene ligand (**18**) with Pd(II) ions resulted in formation of a trigonal prismatic structure (Vasdev et al., 2021). SCXRD data of the BF_4_^−^ salt revealed an eclipsed conformation adopted by the ferrocene units (Figure 3C), with a small variation in the dihedral angle between independent ligands (25–42°).

The self-assembly of twin-armed ferrocene ligands with bidentate coordinating groups has also been investigated. The formation and solid-state structures of dinuclear metallacycles (Figure 3D) assembled from bis(2-pyridyl-1,2,3-triazole) ligand **19** were reported by Crowley and co-workers (Findlay et al., 2018). For both the Ag(I) and Pd(II) metallacycles the ligands were eclipsed, with a slight difference in the dihedral angles observed between the two assemblies (0.9 and 16.2°, respectively). Nitschke and co-workers synthesised isostructural [Fe(II)_4_L_6_]^8+^ twisted parallelogram assemblies from imine and azo ligands **20**/**21** (Plajer et al., 2020). The SCXRD structure of one of these intriguing architectures (Figure 3E) revealed the co-existence of both *syn* (55–67°) and *anti* (118–121°) ligand conformations, demonstrating the ability of chemically identical ligands to occupy inequivalent environments through accessing different conformers. Attempts to oxidise the ferrocene units led to dis-assembly of the parallelogram, and formation of mononuclear [Fe(II)L]^3+^ complexes.

Singleton and co-workers synthesised dipyridyl ligands **22** and **23** (Figure 3F) capable of bond rotation about the amide units (Liu et al., 2020). For **22** the range of favourable conformations was restricted by intramolecular hydrogen bonds between the amide proton and diazaanthracene core. Initially self-assembly of this ligand with Pd(II) resulted in an M_6_L_12_ cube, with an average angle of 97° between the coordinating pyridyl groups in the calculated structure. This species was, however, revealed to be a kinetically trapped intermediate; prolonged heating of the sample led to rearrangement to form a larger M_12_L_24_ cuboctahedron, with the calculated average ligand angle increasing to 120°. In contrast, ligand **23**, with methylated amide units preventing hydrogen bonds across the ligand framework, yielded a simple mononuclear ML_2_ complex (Figure 3F) as a result of increased conformational flexibility.

Recently Yoshizawa and co-workers investigated the dynamics of a Pd_2_L_4_ cage assembled from ditopic ligands with 1,4-naphthylene linkers (**24**) (Tsutsui et al., 2020). Hindered rotation of these units resulted in atropisomeric forms of the ligand. Although this did not inherently affect the relative orientations of the coordinating pyridyl groups, it did lead to an interconverting mixture of 42 virtually isoenergetic cage isomers. Guest encapsulation was found to bias the mixture towards individual isomers, with C_60_ inducing quantitative conversion to the all-*syn* assembly, as shown by SCXRD (Figure 3G). As such, the cage was able to undergo subtle structural modification in response to guest molecules in order to provide a favourable fit.

Alkyl spacers are inherently flexible and are therefore not usually used in MOC ligand design. Their conformational freedom, however, could allow for large amplitude adaptability through interactions with guest molecules. Severin and co-workers successfully synthesised Pd_2_L_4_ cages (Figure 3H) up to 3 nm in length using ditopic metallo-ligands with akyl spacer units (**25**), demonstrating the potential for the controlled incorporation of highly flexible alkyl units into metallo-assemblies (Jansze et al., 2016). Jung, Lee and co-workers obtained SCXRD structures for [Pd_2_L_4_⋑(X^−^)]^3+^ cages (Figure 3I), assembled from a dipyridyl ligand with a flexible disilylethane core (**26**), with a range of encapsulated anions (Lee et al., 2020). The flexibility of the alkyl linker enabled expansion of the capsule to suit the encapsulated anion (Pd···Pd = 8.49 and 9.58 Å for X^−^ = BF_4_^−^ and SiF_6_^−^, respectively). This concept was exploited in the double-cavity [Pd_3_(**27**)_4_⋑(X^−^)_2_]^4+^ architecture, with both homo and hetero-anion adducts formed (Sarada et al., 2020). In this instance each cavity was able to independently adopt an *extended* or *crumpled* shape, dependent on the anion encapsulated (Figure 3J).

**FIGURE 4:**
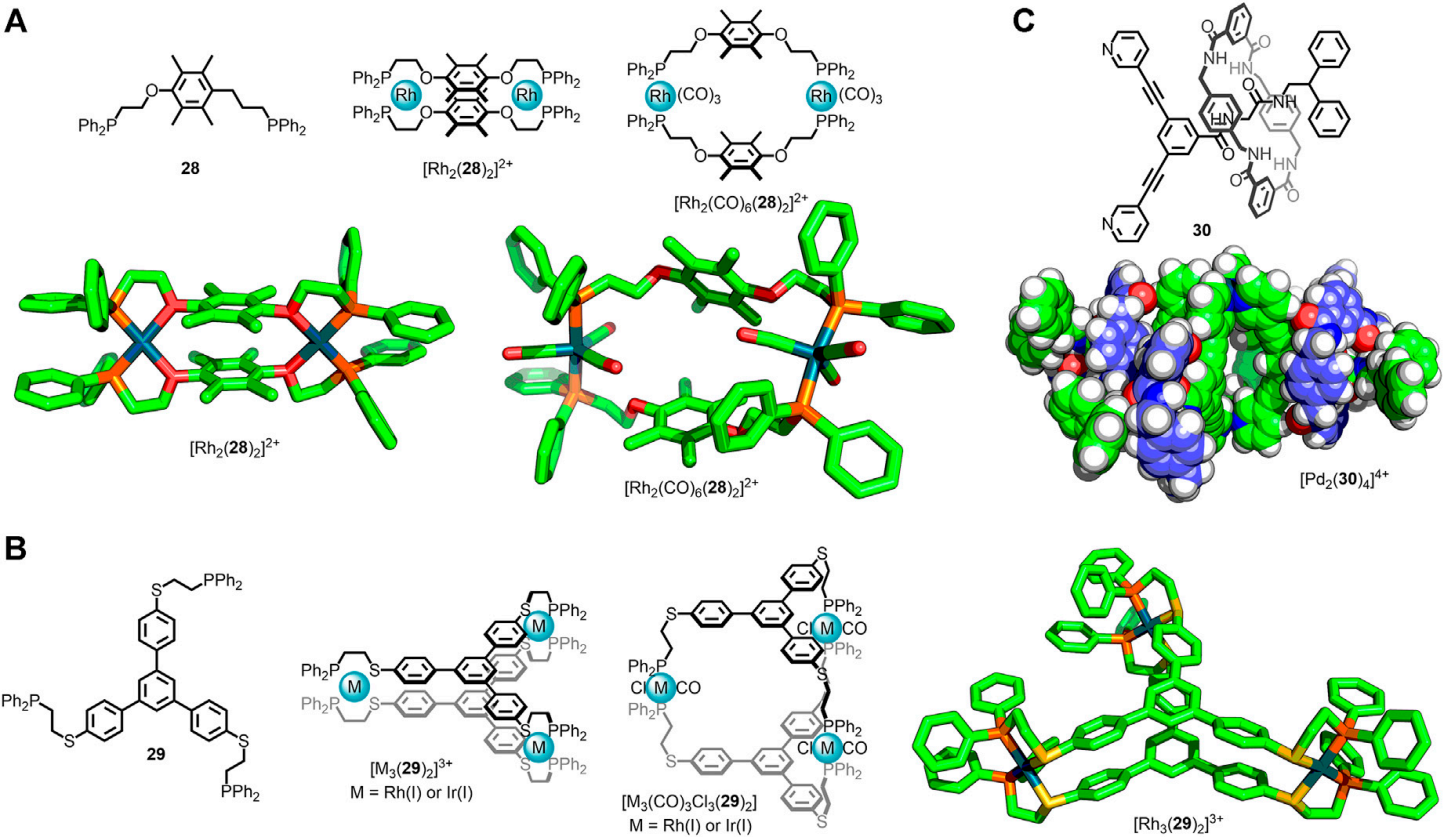
**(A)** Mirkin’s Weak-Link Approach in which a rigid metallacycle precursor, [Rh_2_(**28**)_2_]^2+^, is first formed under thermodynamic control. Subsequent displacement of the ether oxygen donors gave the flexible metallacycle [Rh_2_(CO)_6_(**28**)_2_]^2+^, with ligand **28** coordinated in a bis-monodentate fashion. **(B)** This principle has been applied to MOCs, with flexible cage [M_3_(CO)_3_Cl_3_(**29**)_2_] accessed *via* thermodynamic intermediate [M_3_(**29**)_2_]^3+^ [M = Rh(I) or Ir(I)]. **(C)** Lewis’ [2]rotaxane ligand, **30**, that self-assembled with Pd(II) ions to form a Pd_2_L_4_ cage (molecular model) with dynamic interlocked macrocycle components (shown in blue).

An alternative method to introduce flexible, saturated units into MOCs is to use the Weak-Link Approach (WLA), developed by the Mirkin group (Giannesci et al., 2005). In this methodology kinetically stable metal-organic architectures assembled from flexible ligands are formed *via* ligand exchange from a rigid, thermodynamically-favourable precursor species. This enables access to flexible structures that may not be thermodynamic products and cannot be directly accessed from a combination of ligand and metal nodes. This was first demonstrated with ligand **28** which formed the metallacycle [Rh_2_(**28**)_2_]^2+^, with chelation to the metal centres between the phosphine and ether oxygen (Farrell et al., 1998). The hemilabile oxygen donors could be displaced through addition of competing ligands, such as CO, giving the *expanded* structure [Rh_2_(CO)_6_(**28**)_2_]^2+^ (Figure 4A). The Weak-Link Approach has since been shown to be suitable for the preparation of flexible MOCs (Ovchinnikov et al., 2002). The condensed cage [M_3_(**29**)_2_]^3+^ [M = Rh(I) or Ir(I)] could be *opened* through addition of CO and Cl^−^ ligands, leading to the expanded structure, [M_3_(CO)_3_Cl_3_(**29**)_2_] (Figure 4B).

Mechanically interlocked molecules have been widely investigated for their use as molecular machines due to the high degree of co-conformational freedom between their components afforded by the lack of covalent bonds (Heard and Goldup, 2020). Consequently there has been considerable interest in the development of mechanically interlocked ligands (Lewis et al., 2017) and the use of metal ions/nodes to periodically arrange them (Hoyas Pérez and Lewis, 2020; Wilson and Loeb, 2020). Lewis and co-workers recently reported the synthesis of [2]rotaxane ligand **30** (Figure 4C), wherein one of the stopper units preventing escape of the macrocycle component was based on dipyridyl ligand **9** (Wong et al., 2020). Self-assembly with Pd(II) ions led to the quadruple-stranded cage [Pd_2_(**30**)_4_]^4+^. NMR analysis of this structure revealed a single set of signals for the macrocycle, indicating rapid pirouetting on the NMR timescale. As such the large degree of co-conformational freedom inherent to rotaxane **30** was maintained in the metal-organic assembly, demonstrating the potential for mechanical bonds to be employed as flexible components within MOCs.

## Conclusion and Future Outlooks

The complexity of MOCs that are routinely reported has increased dramatically since preliminary studies in the field over thirty years ago (Pullen et al., 2021). Heteroleptic (Bloch and Clever, 2017), mixed-metal (Li et al., 2015) and low symmetry (Lewis and Crowley, 2020) assemblies towards the development of more sophisticated host systems are becoming more commonplace. Coincident with this, easier access to and improvements within SCXRD and the advancement of computational power for theoretical investigations allow researchers to gain rapid and in-depth analysis of these systems. This has led to some remarkable applications for MOCs, particularly in the area of catalysis. Appreciation of the structural flexibility these systems are capable of, however, is largely overlooked, despite the parallels that are often drawn between MOCs and enzymes. Herein we have taken a brief look at some of the solution-phase, solid-state and computational data available to highlight how significant variations in the structural parameters of MOCs can be. If this flexibility could be harnessed it could open up the doors for developing more sophisticated artificial systems to mimic biological machinery. Steps towards this lofty goal have already been undertaken, with a handful of examples reported of MOCs derived from ligands containing moieties with significant conformational freedom. With continuing investigations into flexible metal-organic assemblies, elucidation of methodologies for their controlled self-assembly and manipulation will lead to increasingly sophisticated systems capable of displaying adaptive behaviour and allow their use in new and exciting applications.
